# Faecal haemoglobin concentrations are associated with all-cause mortality and cause of death in colorectal cancer screening

**DOI:** 10.1186/s12916-022-02724-3

**Published:** 2023-01-24

**Authors:** Lasse Kaalby, Ulrik Deding, Issam Al-Najami, Gabriele Berg-Beckhoff, Thomas Bjørsum-Meyer, Tinne Laurberg, Aasma Shaukat, Robert J. C. Steele, Anastasios Koulaouzidis, Morten Rasmussen, Morten Kobaek-Larsen, Gunnar Baatrup

**Affiliations:** 1 Department of Surgery, Odense University Hospital, 5000 Odense, Denmark; 2 Department of Clinical Research, University of Southern Denmark, 5000 Odense, Denmark; 3 Unit for Health Promotion Research, Department of Public Health, University of Southern Denmark, 6700 Esbjerg, Denmark; 4 Unit for Health Research, Hospital South West Jutland, 6700 Esbjerg, Denmark; 5 Steno Diabetes Centre Aarhus, Aarhus University Hospital, Aarhus, Denmark; 6 GI Section, Minneapolis VA Medical Center and University of Minnesota, Minneapolis, MN 55417 USA; 7 Division of Gastroenterology NYU Langone, New York, NY 10016 USA; 8 Centre for Research into Cancer Prevention and Screening, University of Dundee School of Medicine, Dundee, DD1 9SY UK; 9 Digestive Disease Center, Bispebjerg University Hospital, 2400 Copenhagen, Denmark

**Keywords:** cause of death, colorectal cancer screening, faecal immunochemical test, faecal haemoglobin, mortality, occult blood

## Abstract

**Background:**

Colorectal cancer (CRC) screening reduces all-cause and CRC-related mortality. New research demonstrates that the faecal haemoglobin concentration (f-Hb) may indicate the presence of other serious diseases not related to CRC. We investigated the association between f-Hb, measured by a faecal immunochemical test (FIT), and both all-cause mortality and cause of death in a population-wide cohort of screening participants.

**Methods:**

Between 2014 and 2018, 1,262,165 participants submitted a FIT for the Danish CRC screening programme. We followed these participants, using the Danish CRC Screening Database and several other national registers on health and population, until December 31, 2018. We stratified participants by f-Hb and compared them using a Cox proportional hazards regression on all-cause mortality and cause of death reported as adjusted hazard ratios (aHRs). We adjusted for several covariates, including comorbidity, socioeconomic factors, demography and prescription medication.

**Results:**

We observed 21,847 deaths in the study period. Our multivariate analyses indicated an association relationship between increasing f-Hb and the risk of dying in the study period. This risk increased steadily from aHR 1.38 (95% CI: 1.32, 1.44) in those with a f-Hb of 7.1–11.9 μg Hb/g faeces to 2.20 (95% CI: 2.10, 2.30) in those with a f-Hb ≥60.0 μg Hb/g faeces, when compared to those with a f-Hb ≤7.0 μg Hb/g faeces. The pattern remained when excluding CRC from the analysis. Similar patterns were observed between incrementally increasing f-Hb and the risk of dying from respiratory disease, cardiovascular disease and cancers other than CRC. Furthermore, we observed an increased risk of dying from CRC with increasing f-Hb.

**Conclusions:**

Our findings support the hypothesis that f-Hb may indicate an elevated risk of having chronic conditions if causes for the bleeding have not been identified. The mechanisms still need to be established, but f-Hb may be a potential biomarker for several non-CRC diseases.

**Supplementary Information:**

The online version contains supplementary material available at 10.1186/s12916-022-02724-3.

## Background

Colorectal cancer (CRC) screening with faecal testing significantly impacts CRC mortality sufficiently to affect all-cause mortality [1–8]. The quantitative faecal immunochemical test (FIT) is the most frequently used test [9]. In the Danish CRC screening programme, participants with a positive FIT (faecal haemoglobin concentration (f-Hb) ≥100 ng Hb/mL buffer or ≥20 μg Hb/g faeces) are referred for colonoscopy. 37.0% of these colonoscopies reveal no neoplastic findings that explain the bleeding [10]. This issue is not unique to the Danish screening programme, and the sensitivity of the FIT is often discussed [11]. The presence of haemoglobin in the faeces without an obvious source of bleeding could indicate early-stage non-communicable chronic disease [12]. Recent studies suggest that f-Hb is associated with several seemingly unrelated chronic conditions and causes of death, including cardiovascular disease (CVD), respiratory disease and/or neuropsychological disorders [13–16]. However, these studies are limited by either the use of guaiac-based faecal occult blood test (gFOBT) instead of FIT, the lack of prescription medication associated with gastrointestinal (GI) bleeding as a covariate and/or the lack of individual-level adjustment for confounders. Therefore, we conducted a register-based study investigating the association between f-Hb and both all-cause mortality and cause-specific mortality in a population-based, FIT-tested cohort of participants in the Danish CRC screening programme.

## Methods

### Study population

**Fig. 1:**
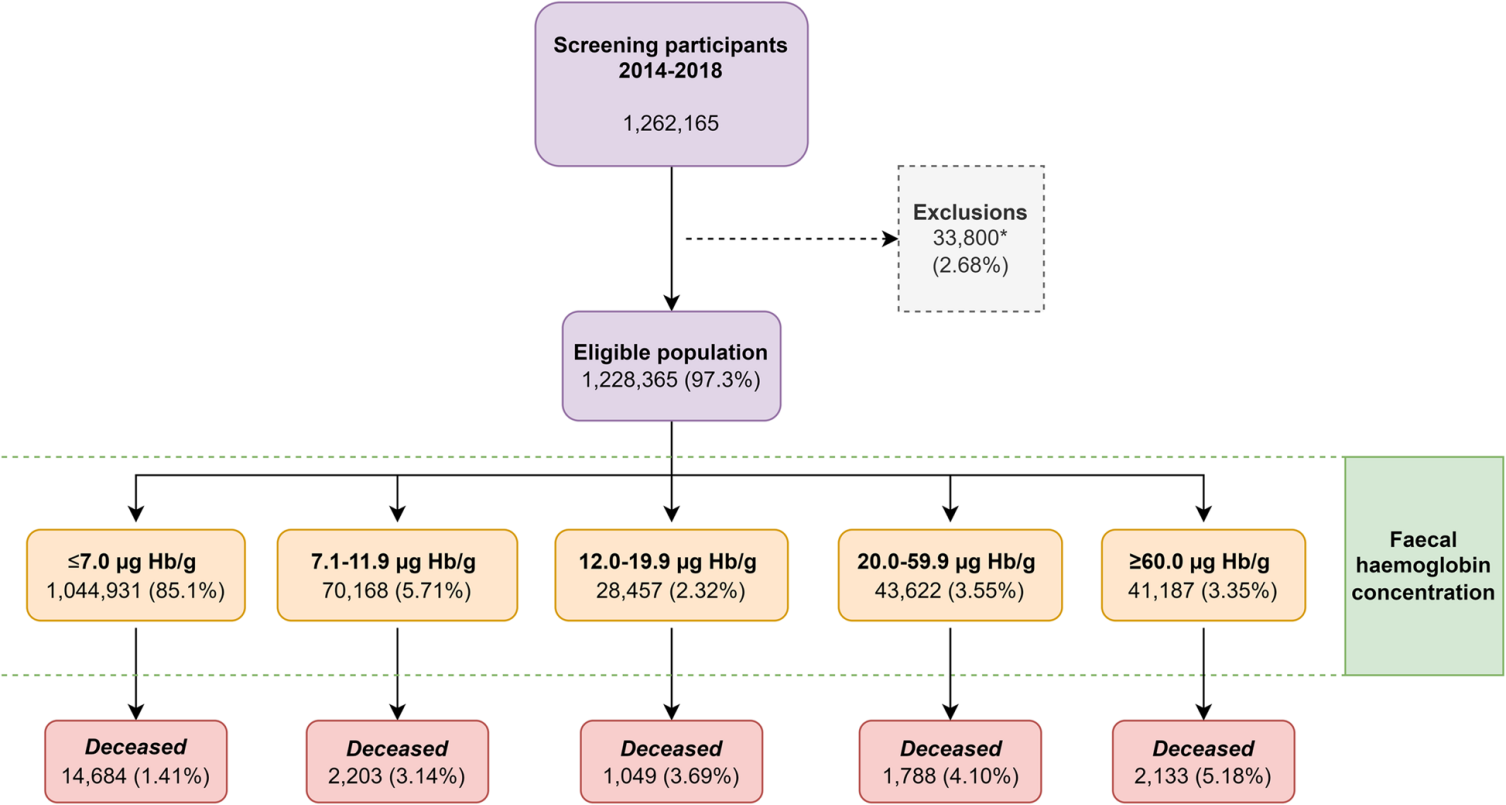
Flow chart of study participants. *Excluded due to missing data

Our study population consisted of all individuals invited to the Danish Colorectal Cancer Screening Program (DCCSP) who had submitted an eligible FIT in the first round of screening. The programme started in 2014, and the last invitations for the first screening round were distributed in 2017. All Danish citizens in the eligible age group (50–74) were invited to participate by submitting a faeces sample. The participant collects one sample of approximately 10 mg of faeces using the OC-Auto Sampling bottle 3 (Eiken Chemical Co, Japan) with the supplied probe and places it in a vial containing 2.0 mL of buffer. The vial is then shipped by prioritized mail to one of five medical laboratories, where it is analysed on the day of receipt. Each medical laboratory covers all FIT assays from their respective healthcare regions and is accredited by the ISO 15189 standard for quality and competencies. If it arrives during the weekend, the test is refrigerated and analysed the coming Monday. The sample remains staple for 7 days at room temperature and 14 days at 2–10°C. The result is sent to the participants within 7 days. If a sample is ineligible for analysis (due to e.g. absence of identification label or inadequate sample material), the patient is sent a new collection kit and a letter explaining the issue. All analyses have been completed using the OC-Sensor Diana instruments (Eiken Chemical Co, Japan). The manufacturer-reported lower limit of detection of the OC-Sensor analyser is 10 μg Hb/g, but a lower limit of 3.8 μg Hb/g faeces has been reported [17, 18]. Mean concentration in unspiked collection tubes has been reported at 2.1 μg Hb/g faeces for the analyser [18]. A quantification limit of 4 μg Hb/g faeces with a variation coefficient of 20% was reported in a recent study [19]. In the DCCSP, the quantitative FIT is considered positive when f-Hb concentration above the positivity threshold of 20 μg Hb/g faeces is detected. All FIT concentrations between 0 and 7.0 μg Hb/g faeces are reported as 7.0 μg Hb/g faeces by the DCCSD. Therefore, all participants with ≤7.0 μg Hb/g faeces were grouped together. In the period 2014–2016, the FIT participation rate was 62.6%, with a positivity rate of 6.9%. 89.1% of those testing FIT positive subsequently underwent colonoscopy [20]. The study baseline was set as the FIT analysis date for each individual. Participants from the first round of screening were followed from baseline to death, migration or end of follow-up (31/12/2018). The flow of participants is presented in Fig. 1.

### Data sources

We conducted an individual-level follow-up for all participants linking national registers on health and population. The study population was identified through the Danish Colorectal Cancer Screening Database (DCCSD), which contains data from the integrated Invitation and Administration Module, the Danish National Patient Register (DNPR) and the National Pathology Register [21]. The DCCSD provides data on screening participation, basic demographics, screening course and screening outcomes. We supplemented this with data from the DNPR on hospital admissions, diagnoses and treatments [22]. Information on prescription medication was obtained from the Danish National Prescription Register [23]. We obtained data on time and cause of death from the Danish Register of Cause of Death (DRCD). Both the underlying and contributing causes registered in the DRCD were used to identify the cause of death to reduce the impact of registration errors [24]. Furthermore, data on socioeconomic factors were obtained by the Danish Education Register [25] and the Income Statistics Register [26].

### Outcome and exposure

We considered all-cause mortality and disease-specific causes of death as individual outcomes. We also included all-cause mortality excluding CRC as an outcome to allow a more nuanced interpretation of the results. The cause of death designation was based on ICD-10 codes. We used the following categories for the cause of death: respiratory disease (excluding lung cancer), diabetes, CVD, other (non-colorectal) cancers and CRC. The definitions are presented in Additional file 1: Table S1. Our main exposure was the f-Hb for all participants presented as an ordinal variable. The concentrations were categorized as ≤7.0 μg Hb/g faeces, 7.1–11.9 μg Hb/g faeces, 12.0–19.9 μg Hb/g faeces, 20.0–59.9 μg Hb/g faeces and ≥60.0 μg Hb/g faeces.

### Covariates

We included a range of baseline covariates using data from the different registers. These were sex, age, highest level of completed education, annual income, conditions suspected of causing GI bleeding (such as inflammatory bowel disease and diverticular disease), prescription medication (e.g. aspirin) and comorbidity. Age was divided into the following categories “*<*55 years”, “55-59 years”, “60-64 years”, “65-69 years” and “>69 years”. The highest level of completed education was defined as “Elementary school”, “Vocational/high school”, “Short-medium length education” and “Long education”. We used person-equated inflation-adjusted income that considered the number of household adults and children of each participant. To allow for income fluctuations, we included the measurement as a 5-year average from baseline and back and categorized participants into quartiles based on income levels. Category 1 is the lowest, and category 4 the highest. We identified participants diagnosed with conditions suspected of causing overt GI bleeding and, therefore a positive FIT, up to 5 years before baseline. The definitions are presented in Additional file 1: Table S2. We included prescription medications known to cause GI bleeding as a covariate for the same reasons. We included all prescriptions collected up to 16 weeks before the FIT analysis date. The medication is detailed in Additional file 1: Table S2.

### Comorbidity

We expected existing comorbidity at baseline to affect both survival and cause of death. We, therefore, employed a standard Charlson Comorbidity Index (CCI) score categorized as “0”, “1” and “>1” for the survival analysis [27]. For the causes of death, we expected that having e.g. CVD at baseline would strongly influence the risk of dying from it. Hence, using only CCI to adjust for comorbidity would lead to an underestimation of the actual effect since there would be no distinction between the condition of interest and the other diseases included in the CCI. Therefore, we created five additional versions of the CCI, one for each of the causes of death. We removed any condition suspected of contributing extensively to the outcome from the CCI and added it as a separate covariate instead. Using diabetes as an example, this allowed us to investigate the association between f-Hb and diabetes mortality while accounting for both the effect of having diabetes and the effect of having other serious diseases at baseline. As a result, we have one covariate for the baseline presence of disease for each of the causes of death, reported as disease present at baseline “Yes” or “No”. The modified CCI scores are reported just like the unmodified version: “0”, “1” and “>1”. The excluded parts are presented in Additional file 1: Table S3.

### Statistics

Descriptive statistics were employed to compare the groups of study participants. Kaplan-Meier curves were used to present mortality and cause of death developments. Cox proportional hazards regression models were used to investigate differences between the exposure groups. The control group was those with a f-Hb of <7.0 μg Hb/g faeces. Results are reported as adjusted hazard ratios (aHRs) or crude hazard ratios (HRs) both with 95% confidence intervals. Both univariate and multivariate analyses were conducted for all outcomes. Log-log plots were used to assess the proportional hazards assumptions. Participants with missing data on one or more covariates were excluded from the analysis. We investigated the presence of effect modification on all outcomes and significant covariates. A sensitivity analysis excluding the participants with a prescription for any anticoagulant medication (ATC-code: B01A) was conducted. All analyses were performed in Stata 17 [28].

## Results

### Characteristics

During the first round of screening in Denmark, 1,262,165 invited residents submitted an eligible FIT sample. We excluded 33,800 (2.68%) due to either missing data or registry errors. The remaining study population comprised 1,228,365 (97.3%) participants, and it was divided into five categories based on the f-Hb. Table 1 demonstrates the observed differences between our exposure groups’ several covariates. Increasing the f-Hb was associated with an increase in the proportion of males, participants who were older and of lower education level and income, had GI bleeding at baseline, were issued prescription medications suspected of causing GI bleeding, had lower education, had lower income and had a higher frequency of comorbidity.

**Table 1:** Characteristics of the study population by faecal haemoglobin concentration

Faecal haemoglobin concentration (μg Hb/g faeces)						
	≤7.0 (*n*=1,044,931)	7.1–11.9 (*n*=70,168)	12.0–19.9 (*n*=28,457)	20.0–59.9 (*n*=43,622)	≥60.0 (*n*=41,187)	Total (*n*=1,228,365)
	*n* (%)	*n* (%)	*n* (%)	*n* (%)	*n* (%)	*n* (%)
Sex						
Female	575,530 (55.1)	36,866 (52.5)	13,943 (49.0)	20,211 (46.3)	17,453 (42.4)	664,033 (54.1)
Male	469,401 (44.9)	33,302 (47.5)	14,514 (51.0)	23,411 (53.7)	23,734 (57.6)	564,362 (45.9)
Age group						
50–55 years	280,312 (26.8)	19,013 (27.1)	5145 (18.1)	7335 (16.8)	6522 (15.8)	318,327 (25.9)
55–60 years	186,618 (17.9)	10,168 (14.5)	4145 (14.6)	5962 (13.7)	5433 (13.2)	212,326 (17.3)
60–65 years	184,096 (17.6)	11,129 (15.9)	5009 (17.6)	7674 (17.6)	7154 (17.4)	215,062 (17.5)
65–70 years	189,363 (18.1)	12,640 (18.0)	6069 (21.3)	9583 (22.0)	9141 (22.2)	226,796 (18.5)
70–75 years	204,542 (19.6)	17,218 (24.5)	8089 (28.4)	13,068 (30.0)	12,937 (31.4)	225,853 (20.8)
GI bleedings at baseline						
Yes	41,655 (4.0)	3466 (4.9)	1769 (6.2)	2732 (6.3)	2749 (6.7)	52,381 (4.3)
No	1,003,266 (96.0)	66,702 (95.1)	26,688 (93.8)	40,890 (93.7)	38,438 (93.3)	1,175,984 (95.7)
Prescription medication						
Yes	525,339 (50.3)	34,709 (49.5)	17,104 (60.1)	26,198 (60.1)	24,302 (59.0)	627,652 (51.1)
No	519,592 (49.7)	35,459 (50.5)	11,353 (39.9)	17,424 (39.9)	16,885 (41.0)	600,713 (48.9)
Educational level						
Elementary school	243,671 (23.3)	19,219 (27.4)	8096 (28.5)	12,502 (28.7)	12,066 (29.3)	295,554 (24.1)
Vocational/high school	474,709 (45.4)	32,477 (46.3)	13,350 (46.9)	20,440 (46.9)	19,522 (47.4)	560,498 (45.6)
Short-medium education	248,235 (23.8)	14,529 (20.7)	5474 (19.2)	8370 (19.2)	7517 (18.3)	284,125 (23.1)
Long education	78,316 (7.5)	3943 (5.6)	1537 (5.4)	2310 (5.3)	2082 (5.1)	88,188 (7.2)
Income quartiles						
1st quantile	247,069 (23.6)	20,339 (29.0)	8955 (31.5)	13,793 (31.6)	13,822 (33.6)	303,978 (24.7)
2nd quantile	259,463 (24.8)	18,725 (26.7)	7176 (25.2)	11,141 (25.5)	10,468 (25.4)	306,973 (25.0)
3rd quantile	266,208 (25.5)	16,883 (24.1)	6456 (22.7)	9770 (22.4)	8961 (21.8)	308,278 (25.1)
4th quantile	272,191 (26.1)	14,211 (20.2)	5870 (20.6)	8918 (20.4)	7936 (19.3)	309,136 (25.2)
History of respiratory disease						
Yes	47,928 (4.6)	3446 (4.9)	1435 (5.0)	2189 (5.0)	1912 (4.6)	56,910 (4.6)
No	997,003 (95.4)	66,722 (95.1)	27,022 (95.0)	41,433 (95.0)	39,275 (95.4)	1,171,455 (95.4)
History of diabetes						
Yes	35,252 (3.4)	2999 (4.3)	1557 (5.5)	2315 (5.3)	2165 (5.3)	44,288 (3.6)
No	1,009,679 (96.6)	67,169 (95.7)	26,900 (94.5)	41,307 (94.7)	39,022 (94.7)	1,184,077 (96.4)
History of cardiovascular disease						
Yes	322,509 (30.9)	24,919 (35.5)	11,475 (40.3)	17,815 (40.8)	17,031 (41.4)	393,749 (32.1)
No	722,422 (69.1)	45,249 (64.5)	16,982 (59.7)	25,807 (59.2)	24,156 (58.7)	834,616 (67.9)
History of other cancers						
Yes	96,989 (9.3)	7067 (10.1)	3505 (12.3)	5560 (12.7)	5261 (12.8)	118,382 (9.6)
No	947,942 (90.7)	63,101 (89.9)	24,952 (87.7)	38,062 (87.3)	35,926 (87.2)	1,109,983 (90.4)
History of colorectal cancer						
Yes	4112 (0.4)	354 (0.5)	189 (0.7)	317 (0.7)	379 (0.9)	5351 (0.4)
No	1,040,819 (99.6)	69,814 (99.5)	28,268 (99.3)	43,305 (99.3)	40,808 (99.1)	1,223,014 (99.6)
Charlson Comorbidity Index score						
0	856,810 (82.0)	55,625 (79.3)	21,188 (74.5)	32,352 (74.2)	30,201 (73.3)	996,176 (81.1)
1	66,437 (6.4)	5186 (7.4)	2597 (9.1)	3745 (8.6)	3498 (8.5)	81,463 (6.6)
>2	121,684 (11.6)	9357 (13.33)	4672 (16.4)	7525 (17.3)	7488 (18.2)	150,726 (12.3)

*Abbreviations*: *f-Hb* faecal haemoglobin concentration, *Hb* haemoglobin, *CRC* colorectal cancer, *GI* gastrointestinal

**Fig. 2:**
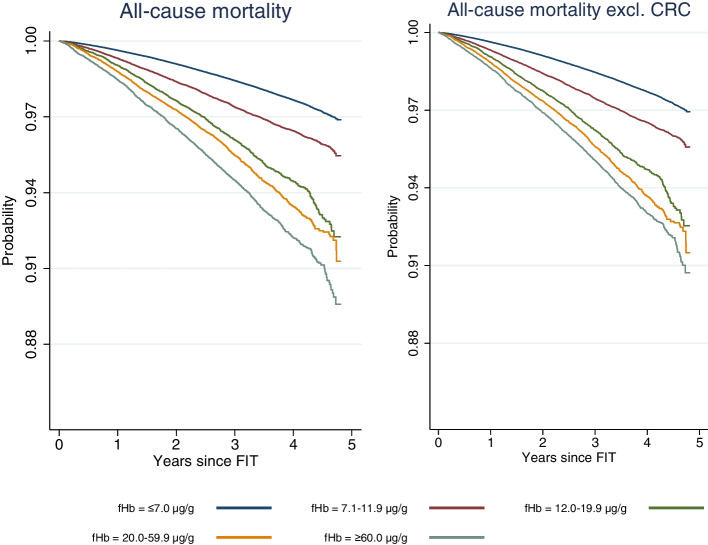
Kaplan-Meier curves on all-cause mortality and f-Hb. Abbreviations: f-Hb, faecal haemoglobin concentration; CRC, colorectal cancer

**Fig. 3:**
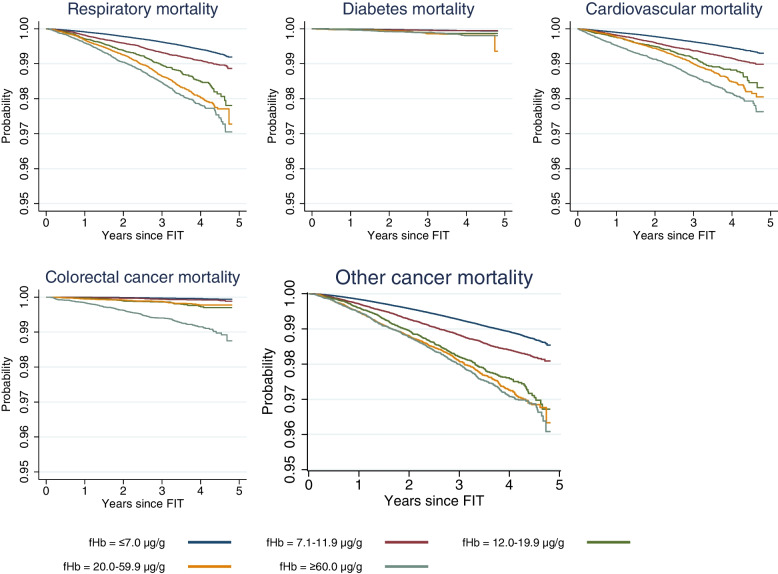
Kaplan-Meier curves on the cause of death and faecal haemoglobin concentration. Abbreviation: f-Hb, faecal haemoglobin concentration

21,857 (1.78%) participants died in the study period, 630 from CRC and 21,227 of causes other than CRC (Table 2). The overall tendency was an increase in the proportion of deaths with increasing f-Hb, which rose steadily from 1.41% of those with a f-Hb ≤7.0 μg Hb/g faeces to 5.18% of those with f-Hb ≥60.0 μg Hb/g faeces. A similar pattern was observed when comparing the f-Hb groups on the probability of dying from the different causes of death, as presented in Figs. 2 and 3. The median follow-up time for the entire population was 2.68 (interquartile range, 1.77–3.67) years with an average follow-up of 2.70 (±1.12) years. Among those who died, the median follow-up was 1.88 (interquartile range, 0.97–2.71) years, with an average follow-up of 1.88 (±1.11) years.

**Table 2:** Distribution of deaths by faecal haemoglobin concentration

Faecal haemoglobin (μg Hb/g faeces)						
Cause of death	≤7.0 (*n*=1,044,931)	7.1–11.9 (*n*=70,168)	12.0–19.9 (*n*=28,457)	20.0–59.9 (*n*=43,622)	≥60.0 (*n*=41,187)	Total (*n*=1,228,365)
	*n* (%)	*n* (%)	*n* (%)	*n* (%)	*n* (%)	*n* (%)
All-cause						
Yes	14,684 (1.4)	2203 (3.1)	1049 (3.7)	1788 (4.1)	2133 (5.2)	21,857 (1.8)
No	1,030,247 (98.6)	67,965 (96.89)	27,408 (96.3)	41,834 (95.9)	39,054 (94.8)	1,206,508 (98.2)
All-cause excl. CRC						
Yes	14,433 (1.4)	2154 (3.1)	1006 (3.5)	1732 (4.0)	1902 (4.6)	21,227 (1.7)
No	1,030,498 (96.6)	68,014 (96.9)	27,451 (96.5)	41,890 (96.0)	39,285 (95.4)	1,207,138 (98.3)
Respiratory disease						
Yes	3600 (0.3)	560 (0.8)	276 (1.0)	508 (1.2)	581 (1.4)	5525 (0.4)
No	1,041,331 (99.7)	69,608 (99.2)	28,181 (99.0)	43,114 (98.8)	40,606 (98.6)	1,222,840 (99.6)
Diabetes						
Yes	338 (0.03)	32 (0.1)	25 (0.1)	46 (0.1)	46 (0.1)	487 (0.04)
No	1,044,593 (99.97)	70,136 (99.9)	28,432 (99.9)	43,576 (99.9)	41,141 (99.9)	1,227,878 (99.96)
Cardiovascular disease						
Yes	3481 (0.3)	522 (0.7)	228 (0.8)	391 (0.9)	516 (1.3)	5138 (0.4)
No	1,041,450 (99.7)	69,646 (99.3)	28,229 (99.2)	43,231 (99.1)	40,671 (98.7)	1,223,227 (99.6)
Other cancers						
Yes	6797 (0.66)	960 (1.4)	451 (1.6)	748 (1.7)	754 (1.8)	9710 (0.8)
No	1,038,134 (99.4)	69,208 (98.6)	28,006 (98.4)	42,874 (98.3)	40,433 (98.2)	1,218,655 (99.2)
Colorectal cancer						
Yes	251 (0.02)	49 (0.1)	43 (0.2)	56 (0.1)	231 (0.6)	630 (0.01)
No	1,044,680 (99.98)	70,119 (99.9)	28,414 (99.8)	43,566 (99.9)	40,956 (99.4)	1,227,725 (99.9)

*Abbreviations*: *f-Hb* faecal haemoglobin concentration, *Hb* haemoglobin, *CRC* colorectal cancer

### Faecal haemoglobin concentration, all-cause mortality and cause of death

**Fig. 4:**
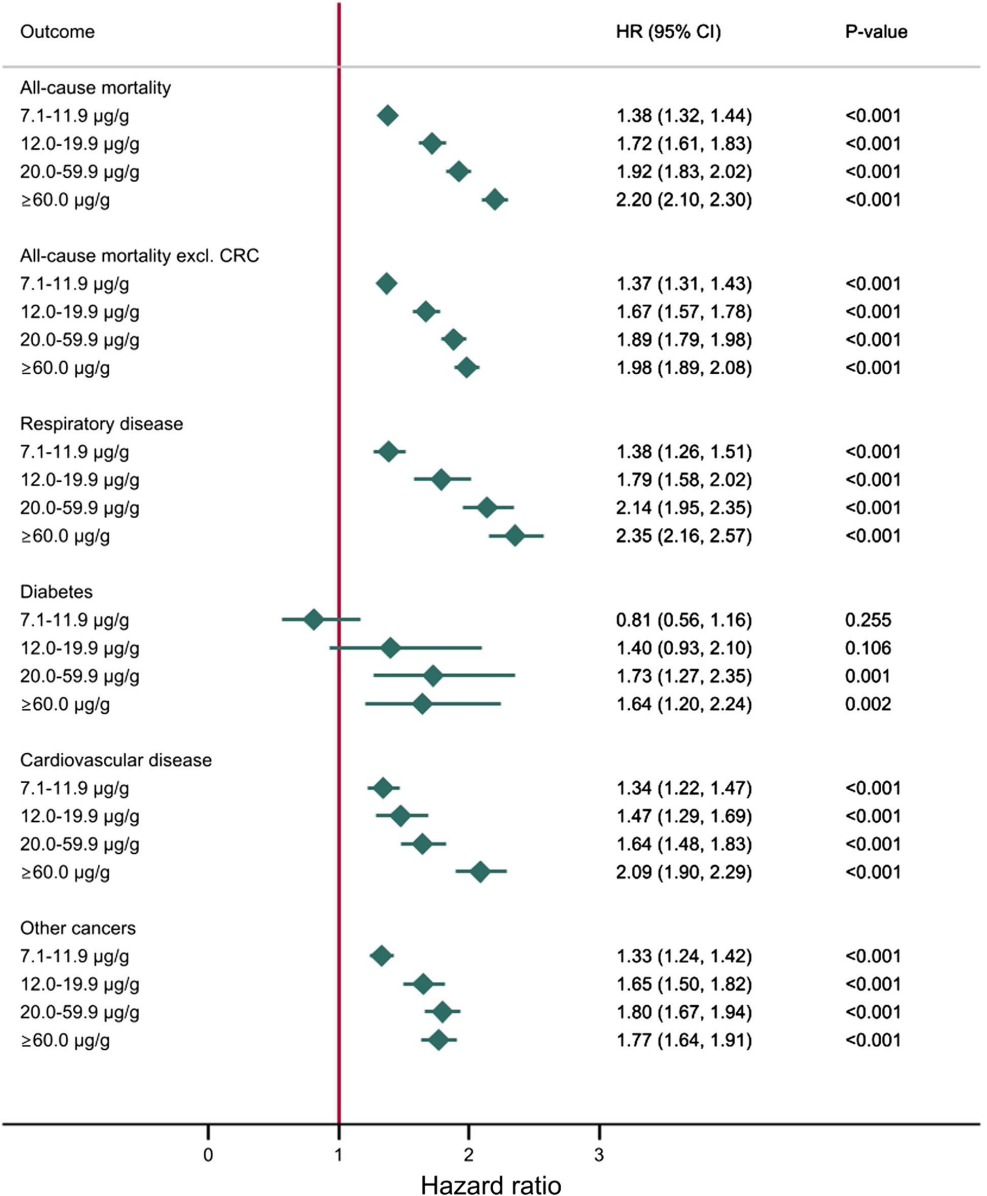
Mortality and faecal haemoglobin. Hazard ratios from multivariate analyses on mortality and f-Hb. Abbreviations: f-Hb, faecal haemoglobin concentration; HR, hazard ratio; CRC, colorectal cancer

The results of our multivariate analyses are presented in Fig. 4. All-cause mortality aHR increased with f-Hb from 1.38 (95% CI: 1.32, 1.44) in those with a *f-Hb* of 7.1–11.9 μg Hb/g faeces to 2.20 (95% CI: 2.10, 2.30) for those with *f-Hb* ≥60.0 μg Hb/g faeces when compared to those ≤7.0 μg Hb/g faeces. Considering all-cause mortality after excluding CRC deaths showed the same increasing trend in aHRs from 1.37 (95% CI: 1.31, 1.43) in those with f-Hb 7.1–11.9 μg Hb/g faeces to 1.98 (95% CI: 1.89, 2.08) in those with f-Hb ≥60.0 μg Hb/g faeces.

A similar trend was seen in the risk of dying from respiratory disease and CVD. For diabetes, the cause-specific mortality increased with increasing f-Hb for the groups ≥20.0 μg Hb/g faeces. However, confidence intervals were wide and overlapping. The risk of dying from other cancers than CRC also increased with increasing f-Hb, from an aHR of 1.33 (95% CI: 1.24, 1.42) at f-Hb 7.1–11.9 μg Hb/g faeces to 1.77 (95% CI: 1.64, 1.91) at f-Hb ≥60.0 μg Hb/g faeces. In the sensitivity analysis, we repeated the multivariate regression without the 17.7% of participants with a prescription for anticoagulant medication. The analysis did not have any significant impact on the results.

For each outcome, we also conducted univariate analyses yielding the same overall conclusion as the multivariate analyses, which, with the exception of diabetes, presented with a more pronounced association to f-Hb. These findings are presented in Additional file 1: Fig. S1. The predictive value of f-Hb for all-cause mortality was further investigated by exploring the margins of the multivariate Cox regression. The results are presented in Additional file 1: Fig. S2.

### Colorectal cancer mortality

**Fig. 5:**
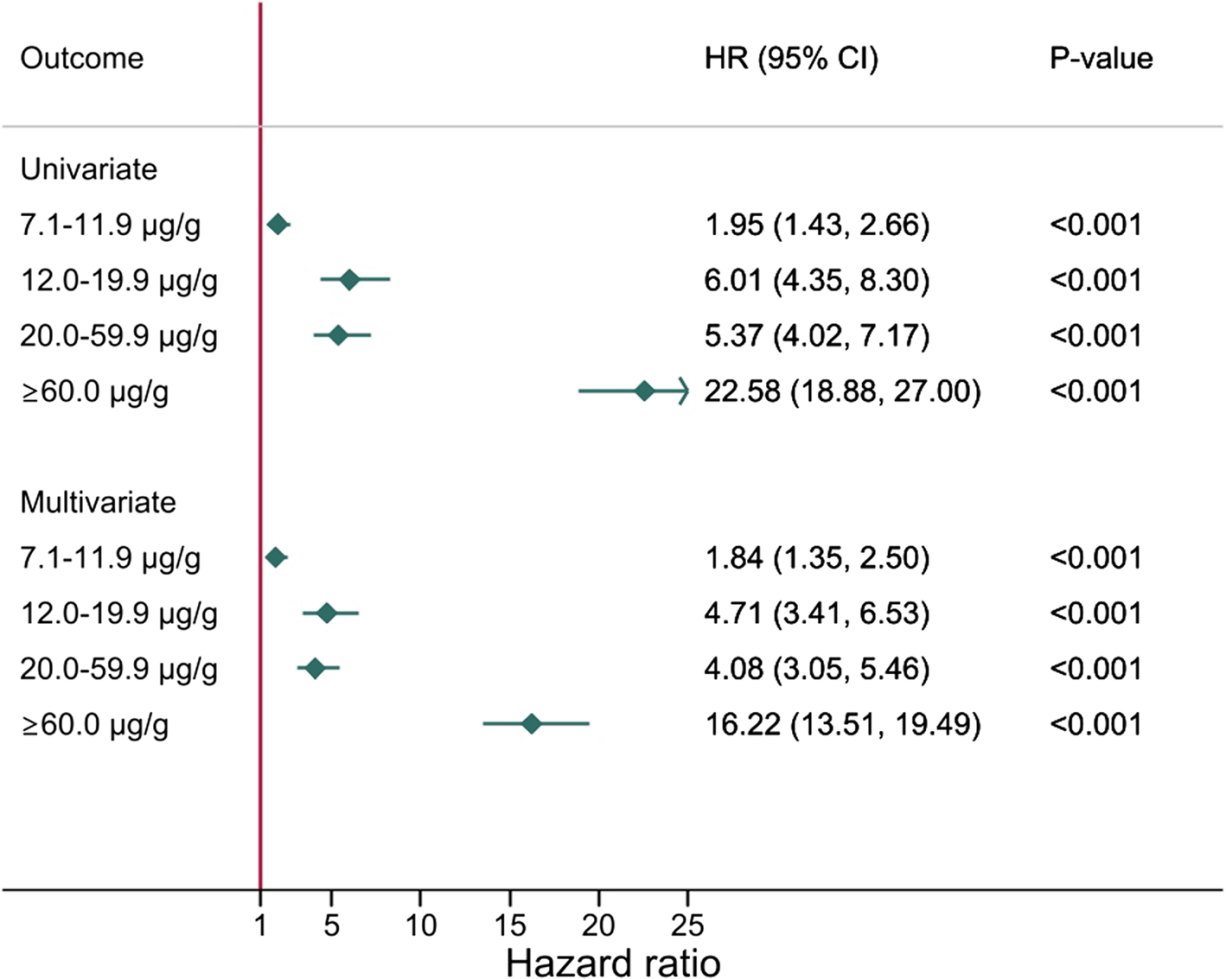
Results from both univariate and multivariate analyses on CRC mortality and f-Hb. Abbreviations: f-Hb, faecal haemoglobin concentration; HR, hazard ratio; CRC, colorectal cancer

Results from the univariate and multivariate analysis are presented in Fig. 5 and show an increase in the risk of CRC death with increasing f-Hb when compared to those with f-Hb ≤7.0 μg Hb/g faeces. Multivariate analysis showed that participants with f-Hb of 7.1–11.9 μg Hb/g faeces had a 1.84 (95% CI: 1.35, 2.50) times higher risk of dying from CRC. This increased to 4.71 (95% CI: 3.41, 6.53) for those with f-Hb of 12.0–19.9 μg Hb/g faeces. For those with f-Hb of 20.0–59.9 μg Hb/g faeces, the risk of dying from CRC was 4.08 (95% CI: 3.05, 5.46) times higher. Participants with a f-Hb of ≥60 μg Hb/g were 16.22 (95% CI: 13.51, 19.49) times more likely to die from CRC than the control group.

## Discussion

Our results indicate that f-Hb is associated with all-cause mortality and seemingly unrelated causes of death in a cohort of screening participants in the Danish national CRC screening program. The significance of our findings is underlined by a significant association relationship between f-Hb and all-cause mortality. For causes of death, this effect was especially notable with CVD and respiratory disease. However, the increased risk of dying from other cancers seems to plateau at f-Hb of ≥20.0 μg Hb/g faeces. In addition, an association between diabetes mortality and f-Hb was observed, although a low number of events and a wide CI reduce interpretability. Since diabetes is a common disease, it is possible that the cause of death in some diabetics may be registered to complications of the disease rather than diabetes itself [29]. Less surprising was the observed relationship between f-Hb and CRC, which was particularly pronounced in the group with the highest f-Hb. However, the increase in aHR appeared to plateau in participants with f-Hb ≥20.0 μg Hb/g faeces, i.e. those just above the Danish positivity threshold. This should be interpreted cautiously as we have a low number of CRC deaths in each category, but one suggested explanation is that this stagnation in mortality around the positivity threshold shows the impact of CRC screening on the mortality curve.

The role of f-Hb in diseases with a systemic inflammatory component represents a growing area of research. A recent review summarizes the hypotheses for the observed patterns, suggesting that elevated f-Hb is an indicator of systemic inflammation and possibly a marker of susceptibility to non-communicable diseases [12]. Recently, Scottish and Danish researchers presented findings that indicated a relationship between f-Hb (measured by gFOBT) and both all-cause mortality and seemingly unrelated causes of death in CRC screening populations with very long follow-up [15, 16]. Korean researchers categorized a FIT-based screening population as either FIT positive or negative, concluding that FIT-positive participants had a higher risk of dying from non-CRC causes, circulatory disease and respiratory disease [30]. Furthermore, in a study from Taiwan, there was a similar dose-response relationship between f-Hb concentration on all-cause mortality and CRC mortality in a cohort of CRC screening participants [31].

Other studies have focused on the relationship between f-Hb and the likelihood of having an underlying non-communicable disease. Japanese researchers have demonstrated an association between FIT positivity and concentrations of glycated Hb in an apparently healthy population [13]. Furthermore, a South Korean study showed increasing incidence rates of diabetes with an increasing number of positive FIT during a 6½-year follow-up period [32]. In a large community-based study, researchers from Taiwan suggested a significantly increased risk of developing and dying from CVD with increasing f-Hb [33]. Lastly, Moon et al. support these findings by showing an increased risk of myocardial infarction, ischemic stroke and all-cause mortality in the FIT-positive population [34]. Other studies have investigated the association between f-Hb and the development of inflammatory diseases. One study presents a 16.0% higher risk of developing rheumatoid arthritis among the FIT positive in a large population of Korean screening participants [35]. Moreover, a Taiwanese study on periodontal disease and f-Hb concentrations reported that participants with a concentration of ≥20.0 μg Hb/g faeces had a 33.0% higher risk of their periodontal disease deteriorating to severe when compared to those with f-Hb concentrations <20.0 μg Hb/g faeces [36]. In another study, the authors found that having a positive FIT result was associated with an increased risk of having psoriasis. The authors highlighted the association between psoriasis and a number of conditions documented to be associated with elevated f-Hb such as metabolic syndrome, dyslipidaemia, obesity, diabetes and hypertension [37]. Moreover, a Taiwanese study has shown an association between metabolic syndrome and elevated f-Hb concentrations [38]. In extension, a Taiwanese study on air pollution and CRC reports that high concentrations of ambient fine particulate matter (known to cause inflammation) increase the risk of having a positive FIT by 11.0% [39]. Scottish researchers found an association between the consumption of medication as a proxy marker for disease and f-Hb. Their findings suggest an increased risk of having heart diseases, diabetes, hypertension and depression if participants have a positive gFOBT result [14]. Our results suggest that several characteristics such as higher age, male sex, lower education and lower income are more common among those with elevated f-Hb concentrations. This is consistent with existing literature [40].

Considering the findings of these studies in the context of our results, there appears to be mounting evidence that f-Hb does indicate the presence of non-communicable diseases and since many of the reported associations have a systemic inflammatory component, the proposed relationship between inflammation and GI bleeding seems plausible. However, the mechanisms underlying these associations remain elusive. An explanation is that the use of medications known to cause GI bleeding, such as oral anticoagulants, aspirin, corticosteroids and non-steroidal anti-inflammatory drugs, could affect the associations presented in this study. However, the inclusion of these drug categories as a confounding factor in the multivariate analysis strengthens the interpretability of our results. This, combined with differing conclusions from recent meta-analyses, reduces our concern about the impact of these drugs on the overall interpretation [41, 42]. Another potentially contributing factor to the elevated f-Hb could be diseases that cause bleeding in the GI tract. The immunochemical response of the FIT should in theory, not detect bleedings originating from the upper GI tract due to the digestion of the haemoglobin proteins. However, FIT-positive participants with a negative colonoscopy have been reported to have a higher risk of some upper GI cancers [43]. While the scientific evidence on this topic is limited and findings incoherent, it cannot be ruled out that upper GI bleedings can affect the FIT measured f-Hb [43, 44]. It is also possible that lower GI bleedings from sources such as haemorrhoids, diverticular diseases and inflammatory bowel disease may impact the reported f-Hb to some degree. While a relationship between false-positive FIT result (positive FIT and negative colonoscopy) and both inflammatory bowel disease and other non-neoplastic findings (such as anal fissures) has been reported, no association was found with diverticulosis and haemorrhoids in the same studies [45, 46]. To mitigate this uncertainty, we accounted for the potential effect of both types of bleeding in our multivariate analyses.

The strengths of our study include a very large population of screening participants, extensive and individual-level adjustment for confounding effects and our ability to adjust for a numerical f-Hb. Limitations include the risk of misclassification in the registrations from the DRCD, which is affected by the input quality. We have addressed this by including contributing causes of death in the analyses and have no indications that misclassification could confound the results to any significant extent. Limited follow-up is another disadvantage of our study, but given the size of our population, the use of time-sensitive analyses and the coherence to other studies with longer follow-up, we do not believe that this affects the overall interpretation of our results. Data on over-the-counter medication is not available in Danish registers and represents another limitation. This may result in some underestimation of the effect medications such as aspirin have on the f-Hb. However, because we were able to adjust for the effect of prescription medication, the impact of this limitation is reduced.

Assuming f-Hb is an effective and practical biomarker for non-communicable diseases, several new pathways focusing on maximizing the diagnostic gain from CRC screening and diagnostic initiatives in other clinical areas could be established. For instance, a study from China suggests using f-Hb to predict complications and survival after R0 gastrectomy [47].

## Conclusions

Our study is the first to investigate the association between f-Hb and mortality in a way that addresses many of the limitations of previous studies. We observed an association between f-Hb and both all-cause mortality and cause-specific mortality. Our results show that individuals with increased f-Hb are more prone to die from non-communicable diseases seemingly unrelated to CRC, including respiratory diseases, CVD and other cancers. In many of these associations, we observed an association between f-Hb and mortality. Overall, these findings emphasize the distinct potential of f-Hb as a biomarker for non-communicable conditions other than CRC and the need for future research in this area.

## Supplementary Information

**Additional file 1:** **Table S1.** The file contains a list of the ICD-10 classification codes used to define cause of death. **Table S2.** A list of diseases, indications and medications suspected of causing GI bleedings and the corresponding ICD-10 classification or ATC codes. These have all been included as covariates. **Table S3.** An overview of the adapted CCI’s used as measures of comorbidity. More precisely the table shows which ICD-10 classifications codes were “removed” from the index and added as a separate variable instead. **Figure S1.** Results from the univariate analysis on mortality outcomes and f-Hb. This provides readers some insight into the impact of adding covariates into the regression analyses. **Figure S2.** Predicted hazard ratios using the margins of the multivariate Cox regression on overall survival.

## Data Availability

The data used in this study have been collected from several different Danish registers and access using secure servers of Statistics Denmark. As we do not own this data, we are not able to make it available ourselves. Given the proper clearances however, others will be able to apply Statistics Denmark for access to the same raw data used in this study.

## Abbreviations

aHR: Adjusted hazard ratio
CCI: Charlson Comorbidity Index
CRC: Colorectal cancer
CVD: Cardiovascular disease
DCCSD: The Danish Colorectal Cancer Screening Database
DNPR: The Danish National Patient Register
DRCD: The Danish Register of Cause of Death
f-Hb: Faecal haemoglobin concentration
FIT: Faecal immunochemical test
gFOBT: Guaiac faecal occult blood test
GI: Gastrointestinal
HR: Hazard ratio
