# Supplementary material for: Faecal haemoglobin concentrations are associated with all-cause mortality and cause of death in colorectal cancer screening
Source: BMC Med. 2023 Jan 24;21:29. doi: 10.1186/s12916-022-02724-3 (PMC9872406; doi:10.1186/s12916-022-02724-3)

**Supplementary material**

| **Table S1. Causes of death and corresponding ICD-10 classification** | |
| --- | --- |
| **Cause of death** | **ICD-10 classification** |
| Respiratory disease | J00-J99 |
| Diabetes | E10-E14 |
| Cardiovascular disease | I00-I99 |
| Other cancers | C00-C17, C21-C99 |
| Colorectal cancer | C18-C20 |

**Abbreviations:** ICD, International Classification of Disease.

| **Table S2. Diseases, indications and medications suspected of contributing to gastro-intestinal bleeding** | |
| --- | --- |
| **Diseases and indications** | **ICD-10 classification** |
| Haemorrhage of anus and rectum | K625-K626 |
| Inflammatory bowel disease | K50-K52 |
| Diverticular disease | K57 |
| Haemorrhoids | I84 |
| Colorectal fissures | K60 |
| Gastrointestinal ulcers | K25-K28 |
| Gastritis | K29 |
| **Medications** | **ATC code** |
| All Antiplatelet agents, parenteral and oral anticoagulants | B01A |
| Corticosteroids | H02, A07EA |
| All non-steroidal anti-inflammatory drugs | M01A |

**Abbreviations:** ICD, International Classification of Disease; ATC, Anatomical Therapeutic Chemical.

| **Table S3. Modified versions of Charlson Comorbidity Index** | |
| --- | --- |
| **Index versions** | **ICD-10 codes not included** |
| Non-respiratory disease CCI | J00-J199 |
| Non-diabetes CCI | E10-E14 |
| Non-cardiovascular disease CCI | I00-I99 |
| Non-other cancers CCI | C00-C17, C21-C99 |
| Non-colorectal cancer CCI | C18-C20 |

**Abbreviations**: ICD, International Classification of Disease; CCI, Charlson Comorbidity Index.

**Supplementary Figure S1. Univariate analyses on mortality and faecal haemoglobin concentration**


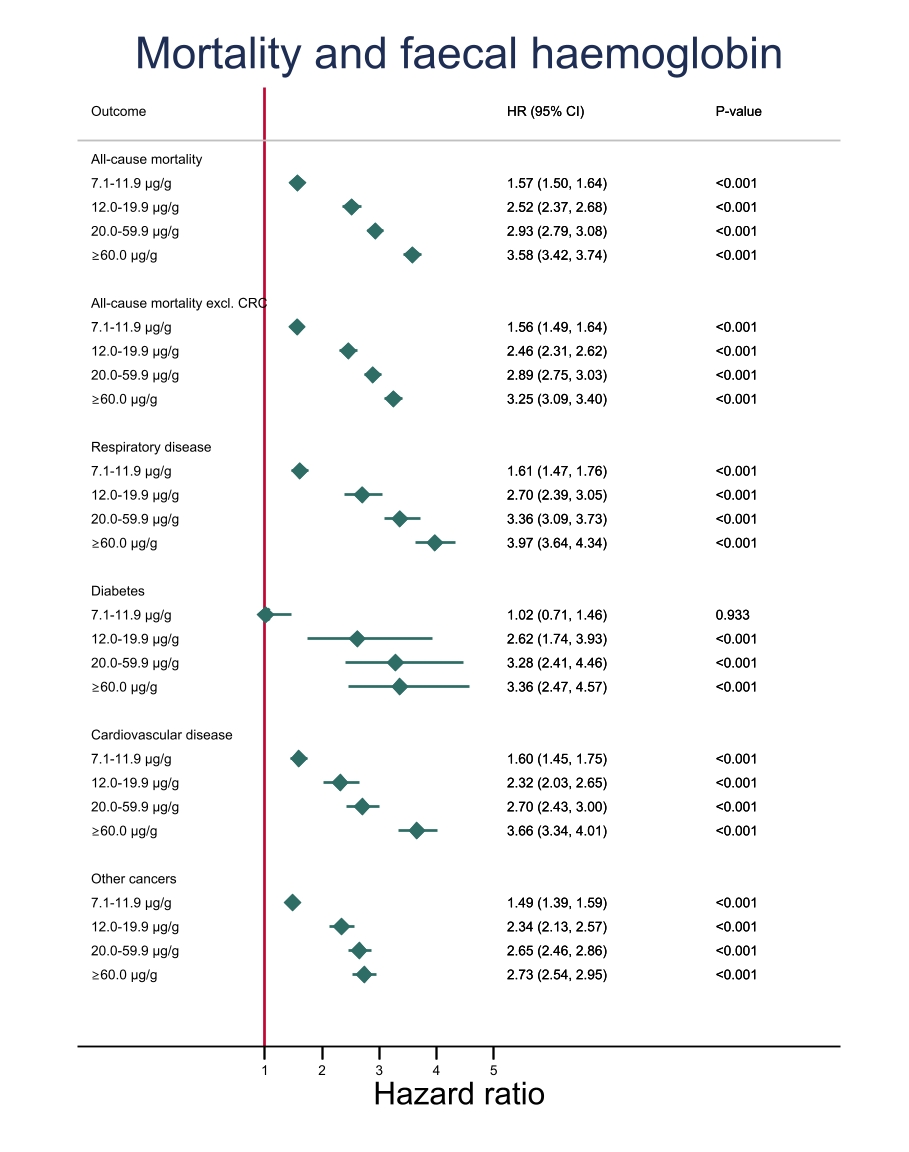


**Abbreviations:** HR, Hazard Ratio; CRC, colorectal cancer.

**Supplementary Figure S2:** ­ Predicted hazard ratios using the margins of the multivariate cox-regression on overall survival. The predicted hazard ratio of each of the f-Hb categories is presented below. Overall, the model confirms that f-Hb does appear to predict all-cause mortality.


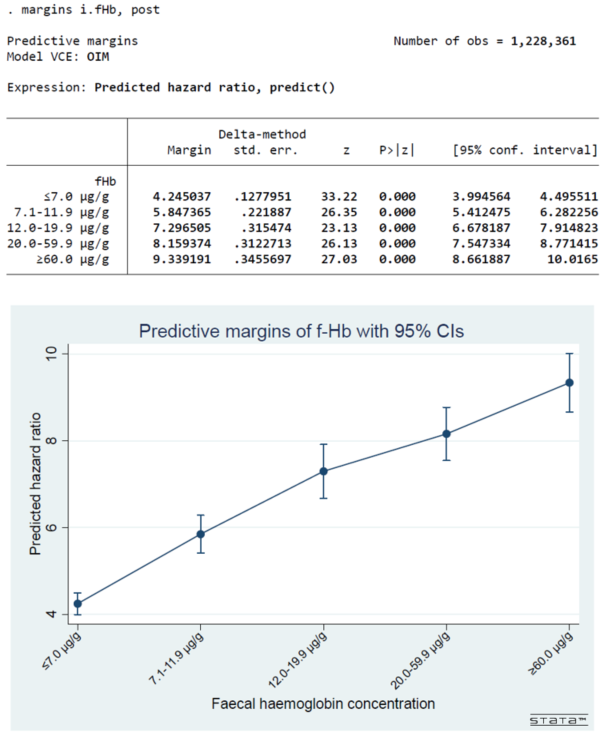

Supplement: Supplementary file 1 — Additional file 1: Table S1. The file contains a list of the ICD-10 classification codes used to define cause of death. Table S2. A list of diseases, indications and medications suspected of causing GI bleedings and the corresponding ICD-10 classification or ATC codes. These have all been included as covariates. Table S3. An overview of the adapted CCI’s used as measures of comorbidity. More precisely the table shows which ICD-10 classifications codes were “removed” from the index and added as a separate variable instead. Figure S1. Results from the univariate analysis on mortality outcomes and f-Hb. This provides readers some insight into the impact of adding covariates into the regression analyses. Figure S2. Predicted hazard ratios using the margins of the multivariate Cox regression on overall survival. [file 12916_2022_2724_MOESM1_ESM.docx]
